# Rural‐urban disparities in Alzheimer's Disease and Related Dementia: The role of state‐level nurse practitioner policies

**DOI:** 10.1002/alz70860_106204

**Published:** 2025-12-23

**Authors:** Nasim Ferdows

**Affiliations:** ^1^ Northeastern University, Boston, MA, USA

## Abstract

**Background:**

With rising life expectancy and a higher share of older adults in rural areas, ADRD rates are expected to increase in these communities. However, limited access to primary care services may contribute to underdiagnosis, particularly in rural areas with physician shortages. State‐level restrictive nurse practitioner (NP) policies further exacerbate access challenges, limiting the availability of providers who can diagnose and manage ADRD. This study examines ADRD trends stratified by state NP practice authority and across multiple datasets, including one requiring medical diagnosis and healthcare access and another that is free from clinical assessment.

**Method:**

We analyzed ADRD trends using three national datasets. County‐level mortality data from CDC (1999–2022) were used to compare rural‐urban mortality rates. Medicare fee‐for‐service (FFS) claims (2013–2020) provided ADRD prevalence estimates based on medical diagnoses requiring provider access. Health and Retirement Study (HRS) data (2010–2022) estimated ADRD prevalence using cognitive assessments, independent of healthcare access. Rurality was classified using county designations, and states were stratified by NP practice authority (full, reduced, and restricted practice) to assess how state‐level policies shape access to ADRD diagnosis in rural communities.

**Result:**

ADRD mortality rates were slightly higher in rural areas compared to urban areas, with no significant differences between rural‐adjacent and rural‐nonadjacent counties in recent years. Medicare claims data showed lower ADRD prevalence in rural areas. In contrast, HRS cognitive assessments revealed higher ADRD prevalence in rural areas. Stratification by NP practice authority showed that states with restricted NP practice had lower ADRD prevalence in Medicare claims data, particularly in rural communities. The difference was less pronounced in full‐practice states, where NPs have independent authority to diagnose and treat ADRD.

**Conclusion:**

Lower ADRD rates in rural Medicare claims data likely reflect limited healthcare access rather than true differences in disease burden. HRS cognitive assessments indicate higher ADRD prevalence in rural areas, suggesting underdiagnosis in claims‐based data. Restrictive NP policies further limit access, particularly in rural communities. Expanding NP practice authority could improve ADRD diagnosis and care, reducing rural disparities.

